# Retraction Statement: Novel NAD‐independent *Avibacterium paragallinarum*: Isolation, characterization and molecular identification in Iran

**DOI:** 10.1002/vms3.1257

**Published:** 2023-09-15

## Abstract

Beiranvand, S., Piri‐Gharaghie, T., Dehganzad, B., Khedmati, F., Jalali, F., AsadAlizadeh, M., & Momtaz, H. (2022). Novel NAD‐independent Avibacterium paragallinarum: Isolation, characterization and molecular identification in Iran. Veterinary Medicine and Science, 8, 1157– 1165. https://doi.org/10.1002/vms3.754. The above article, published online on 19 February 2022 in Wiley Online Library (wileyonlinelibrary.com), has been retracted by agreement between the journal's Editor‐in‐Chief, Dr Gayle Hallowell, and John Wiley & Sons Ltd. The retraction has been agreed following concerns raised that a number of figures within the results section were not original and had been used without permission from the copyright holder. The authors were asked to provide their original data for review by the editorial team to corroborate their results. The editorial team were unable to verify the data provided and, as a result, have concerns about the conclusions of the article. The journal is therefore issuing this retraction.

Beiranvand, S., Piri‐Gharaghie, T., Dehganzad, B., Khedmati, F., Jalali, F., AsadAlizadeh, M., & Momtaz, H. (2022). Novel NAD‐independent Avibacterium paragallinarum: Isolation, characterization and molecular identification in Iran. Veterinary Medicine and Science, 8, 1157– 1165. https://doi.org/10.1002/vms3.754. The above article, published online on 19 February 2022 in Wiley Online Library (wileyonlinelibrary.com), has been retracted by agreement between the journal's Editor‐in‐Chief, Dr Gayle Hallowell, and John Wiley & Sons Ltd. The retraction has been agreed following concerns raised that a number of figures within the results section were not original and had been used without permission from the copyright holder. The authors were asked to provide their original data for review by the editorial team to corroborate their results. The editorial team were unable to verify the data provided and, as a result, have concerns about the conclusions of the article. The journal is therefore issuing this retraction.

